# Latent structure of secondary traumatic stress, its precursors, and effects on people working with refugees

**DOI:** 10.1371/journal.pone.0241545

**Published:** 2020-10-30

**Authors:** Marko Živanović, Maša Vukčević Marković

**Affiliations:** 1 Department of Psychology, Institute of Psychology, University of Belgrade, Belgrade, Serbia; 2 Psychosocial Innovation Network, Belgrade, Serbia; Technion Israel Institute of Technology, ISRAEL

## Abstract

The study aims to examine the latent structure of secondary traumatic stress (STS), its precursors, and the psychological effects of it on the population of service providers working with refugees passing through the Balkan route. A total of 270 service providers (57% female) of different professional backgrounds working directly with refugees took part in the study. Participants were assessed for STS using the Secondary Traumatic Stress Scale, the extent of secondary exposure to trauma (i.e., clients’ traumatic experiences from the countries of origin and travel that were communicated to them directly), depression, anxiety, and quality of life. Comparisons of several confirmatory factor analyses following prominent PTSD conceptualizations showed that the model with three relatively distinct but highly correlated factors–intrusion, avoidance, and the blend of negative alterations in cognitions, mood, and reactivity (NACMR), had the best fit. STS has been shown to be positively correlated both with the amount of different traumatic experiences that were communicated to them as well as with the specific content of those experiences. Path analysis showed that the amount of secondary exposure to the clients’ traumatic experiences during travel, but not in the country of origin, had exclusive relationships with all three factors of STS. NACMR demonstrated direct effects on anxiety and depression symptoms, while intrusions exhibited a direct effect on anxiety-related symptomatology only. The avoidance factor did not have any independent direct effects on anxiety or depression. Finally, the effects of STS factors on quality of life were fully mediated by an increase of depression-related symptomatology. Results provide evidence on the latent structure of the STS which partially deviates from the prominent models of PTSD thus questioning the isomorphism of two constructs on the empirical level. Additionally, findings provide insights on the cascade of events that make professionals working with traumatized people especially vulnerable to STS and broader psychological distress.

## Introduction

Practitioners involved in helping professions are often working with vulnerable populations and are, as such, exposed to *secondary traumatic stress (STS)* or *secondary traumatization*; a condition that results from helping or wanting to help traumatized or suffering individuals [1–3] and which mimics symptoms of post-traumatic stress disorder (PTSD) [4]. Due to the conceptual similarities, the majority of models of secondary trauma rely on PTSD nomenclature and include similar components of both exposure to traumatic content and symptomatology structure [5]. Thus, since the Statistical Manual of Mental Disorders, Fourth Edition (DSM-4) [6] defines Criterion A1 as exposure to the event, (“the person experienced, witnessed, or was confronted with an event or events that involved actual or threatened death or serious injury, or a threat to the physical integrity of self or others”), secondary traumatization is expected and explored among practitioners working with clients who meet Criterion A1, mainly traumatized children, victims of violence, and hospitalized patients [5, 7–9]. Accordingly, previous studies were mainly focused on social workers [10–14] and health professionals–clinical psychologists, physicians, nurses, and midwives [7, 12, 15–20]. Clinician’s responses to exposure to the traumatic experiences of the clients, representing Criteria A2 (“the person’s response involved intense fear, helplessness, or horror”) was; however, generally overlooked, implying that secondary exposure to traumatic content is distressing enough to lead to clinical symptomatology [5]. Changes in Statistical Manual of Mental Disorders–fifth edition (DSM-5) [21] related to Criteria A, eliminated subjective reaction, while exposure was expanded to include the wider scope of events that qualify as traumatic, including “experiencing repeated or extreme exposure to aversive details of the traumatic event(s) (e.g., first responders collecting human remains: police officers repeatedly exposed to details of child abuse)”. This change qualified secondary exposure as a traumatic event *per se* [21] and called into question whether the separation of STS from PTSD was still needed [22].

According to DSM-4 [6], PTSD’s symptomatology structure is grouped around three factors including symptoms of re-experiencing (intrusions), avoidance, and arousal. However, changes introduced in DSM-5 [21] suggested four clusters of symptoms: intrusions which remained almost intact, alterations in arousal and reactivity which kept most of the DSM-4 arousal symptoms and added irritable behavior or angry outbursts and reckless or self-destructive behavior, while the avoidance cluster was divided into two clusters: avoidance and persistent negative alterations in cognitions and mood (NACM) [21]. Previous studies exploring empirical clustering of PTSD symptoms produced ambiguous findings, suggesting several models to be the best representation of the PTSD factor structure. Three factorial models rely on DSM-4 and suggest intrusions, avoidance, and arousal clusters of symptomatology [6]. Four factorial models rely either on DSM-5 clusters of symptomatology [21] or suggest a separate factor consisting of general psychological distress which is a combination of several symptoms of numbing and hyperarousal, as proposed by the Dysphoria model [23, 24]. Meta-analysis relying on DSM-4-based PTSD literature indicated that four-factor models comprised of intrusions, avoidance, hyperarousal, and dysphoria factors appeared to have a better fit when compared to one to three-factorial models [25]. The five-factor *Dysphoric Arousal* model [26], includes clusters of intrusion, avoidance, and NACM, while separates the DSM-4's arousal cluster into two factors—anxious arousal and dysphoric arousal. The six-factor *Anhedonia* model suggests differentiation within the NACM factor into negative affect and anhedonia [27] while the *Externalizing Behaviors* (EB) model [28] proposes an additional factor capturing aggressive and self-destructive reckless behavior, suggesting a six-factor model. Finally, the seven-factor model suggests the following relatively distinct clusters of symptoms to represent PTSD: intrusion, avoidance, dysphoric arousal, anxious arousal, externalizing behavior, negative affect, and anhedonia [27].

In the light of the changes in DSM-5, assuming that STS mimics symptoms of post-traumatic stress disorder, it remains unclear whether current conceptualizations of PTSD and STS and the latent structures of their operationalizations remain comparable.

The Secondary Trauma Stress Scale (STSS) represents one of the most prominent operationalizations of STS. This instrument relies on the DSM-4 conceptualization of PTSD and includes three clusters of symptoms referring to intrusion, avoidance, and arousal. The authors of the scale have shown the STSS to be a psychometrically sound measure, with an internal consistency coefficient of .93 for the total scale, and for the intrusion, avoidance, and arousal subscales of .83, .89, and .85, respectively [11]. Convergent, discriminant, and factorial validity were also confirmed, with confirmatory factor analysis using structural equation modeling, which showed adequate three-factor model fit [11]. Subsequent studies provided further confirmation of good psychometric properties, with a Cronbach’s alpha coefficient for the whole scale ranging from .89 to .94 [10, 12, 13, 15, 16, 18, 19, 29]. The validity of the scale was also confirmed, showing that personal history of trauma [14], exposure to trauma work [14–16], and different types of work-related stress [10, 29] were positively associated with STSS measures, while interpersonal and social support [12, 16], work satisfaction [16], sense of role competence [13], professional experience [14], and self-care [12] were negatively associated with the same measures.

A recent study examined the latent factor structure of the Secondary Traumatic Stress Scale. In order to make it more consistent with changes introduced in DSM-5 PTSD nomenclature, a few changes were made. Three items referring to D3, D4, and E2 diagnostic criteria were added, while two items referring to D1 and E1, were modified. However, the authors concluded that the 7-factor hybrid model, comprised of intrusion, avoidance, negative affect, anhedonia, externalizing behavior, anxious arousal, and dysphoric arousal factors had an excellent fit [22], which successfully replicated that of the seven-factor model of PTSD symptomatology [27].

In order to develop an instrument for the assessment of STS based on DSM-5 nomenclature, some new efforts were made. Thus, Weitkamp and colleagues [4] developed an instrument for STS assessment based on the DSM-5 conceptualization of this phenomenon—the Questionnaire for Secondary Traumatization (FST). However, the examination of the latent structure of this instrument did not result in dimensions of STS that would be comparable to the DSM-5-based concepts of this syndrome, and some of the factors obtained included only one item [4]. Finally, it should be noted that information on the external validity of the FST is still lacking [4].

In sum, there were just a few studies trying to reexamine STS’s clusters of symptoms, and the conclusions they provided were inconsistent. Two-factor [20], three-factor [11, 30], four-factor [31], and seven-factor symptom-grouping [22] were suggested. Previous studies raised a question of whether the replicability of PTSD structure could be dependent on the specific sample used in the study [4]. This pointed out a dilemma regarding whether all service providers that participated in the previous studies are faced with clients/patients who meet Criteria A on a daily basis, a precondition for PTSD and, consequently, STS related difficulties. According to DSM-5 “a *life-threatening illness or debilitating medical condition is not necessarily considered a traumatic event*,*”* yet traumatic event includes medical incidents that involve sudden or catastrophic events (e.g., waking during surgery, anaphylactic shock) and repeated or extreme exposure to aversive details of the traumatic events. Hence, inclusion criteria for service providers who are at risk of secondary traumatic stress used in previous studies, mainly health professionals—physicians, nurses, and midwives, could be one of the reasons for inconsistent findings on expected similarities between PTSD and STS latent structure. Thus, it can be concluded that further evidence based on service providers working with clients/patients who unambiguously meet Criteria A is needed in order to enable theoretical interpretation and understanding of inconsistent empirical findings.

### Current study

Practitioners working with refugees and migrants coming from the Middle East and Africa are on a daily basis faced with people who have faced numerous traumatic experiences and human rights violations including torture, violent death of loved ones, forced mobilization, lack of food, water, or shelter, beatings, problems with smugglers, isolation, poverty, etc. [32–44], experiences out of which the majority can be classified as traumatic [21]. Previous studies demonstrated the negative impact of these events on mental health [32, 34, 35, 38, 39, 42, 44–48], as well as increased PTSD and depression related difficulties among this population [37, 41, 42, 49]. In addition to being secondarily exposed to terrifying human suffering, their work-related difficulties often include uncertainty and lack of resources and systemic support. Moreover, since working with refugees often involves emergency responses without enough time for preparation, they often lack the proper training and supervision needed for working in emergency, humanitarian context, or with traumatized or vulnerable individuals. Hence, practitioners working with refugees who unequivocally meet DSM-5 Criteria A, are exposed to risks for secondary traumatic stress and should be considered a priority when exploring this phenomenon [50, 51]. Surprisingly, this matter only started getting more attention recently, and only a few studies have explored secondary traumatization among service providers working with refugees [50–52].

The main aim of this study is to examine the latent factor structure of Secondary Traumatic Stress (STS) using prominent Posttraumatic Stress Disorder (PTSD) nomenclature in the sample of service providers working with refugees during refugee crises. Moreover, psychometric evaluation of STSS, and validity of the scale was tested through its relationships with secondary exposure to traumatic content, measures of symptomatology including anxiety and depression, and satisfaction with the quality of different aspects of personal and professional life. In addition to potential theoretical implications leading to a better understanding of STS and interpretation of previously suggested models, in the light of the current refugee crises all over the world, empirical evidence is urgently needed in order to enable the creation of recommendations for diagnosis, proper treatment and prevention mechanisms among professionals and volunteers providing services to those in need worldwide [5].

## Method

### Participants

A total of 270 participants (57% female), ages ranging from 18 to 67 (*M* = 33.66, *SD* = 9.58) of different professional backgrounds working directly with refugees volunteered to participate in the study. The selection of participants was done based on pre-established cooperation with governmental and non-governmental institutions, agencies, and organizations and selection criteria for participation was at least one-month engagement in the provision of services to refugees that include direct contact. The majority of participants finished university (73.0%), 21.5% were high school educated, while 5.5% were students. On average, at the time of assessment participants had worked with refugees for 34.68 months (*SD* = 70.73) and were providing different types of services and assistance–professional translators (e.g. Arabic, Farsi, Kurdish, Urdu, Pashto) providing cultural mediation/translation (12.2%), lawyers providing legal assistance and representation (18.2%), psychologists providing psychological support (19.3%), medical doctors/technicians providing medical aid (2.2%), as well as people of different educational backgrounds engaged in either delivering educational, occupational, and recreational (EOR) workshops and activities (23.7%) or providing food and non-food items (8.1%), and staff working in the asylum, receptive, and transit centers across Serbia (16.3%) primarily engaged in the provision of information, accommodation, and referrals. Twenty-two percent of participants reported working in direct contact with beneficiaries up to 2 days a week, 34.4% reported having contact between 2 and 4 days a week, while 43.7% had a full-time contact with refugees and migrants. The vast majority of beneficiaries with whom participants interact are refugees, migrants and asylum seekers from Middle Eastern countries (e.g. Syria, Iraq, Afghanistan, Iran) and African countries (e.g. Morocco, Algeria, Somalia).

### Instruments and measures

#### Secondary Traumatic Stress Scale (STSS)

The STSS [11] is one of the most widely used instruments for assessment of the effects of secondary exposure to trauma. It is a 17-item instrument initially designed to measure the negative effects of social work practice with traumatized populations. Relying on DSM-4, this scale consists of three subscales that measure intrusion, avoidance, and arousal symptoms that are associated with indirect exposure to traumatic events that happened in the course of one’s professional duties and relationships with traumatized clients [11]. Participants were asked to indicate how often they had experienced different types of symptoms over the past seven days. The responses were given on a 5-point scale, ranging from 1 (never) to 5 (very frequently). In line with the authors’ guidelines, scores of 27 or below are interpreted as little or no STS, scores 28–37 as mild STS, scores 38–43 as moderate STS, scores 44–48 as high STS, while scores of 49 and above are interpreted as severe STS.

#### Harvard Trauma Questionnaire, Part I (HTQ)

Secondary exposure to traumatic experiences was assessed using the Harvard Trauma Questionnaire, Part I (HTQ) [53], a cross-culturally validated and widely used check-list for measuring torture and trauma. For the purposes of this research, HTQ Part I, consisting of 64 traumatic experiences refugees could be faced with in their countries of origin [42, 53] was adapted for use with service providers, together with the **Stressful Experiences in Transit Questionnaire–Short Form (SET-SF)** which includes a list of 19 traumatic experiences refugees could be exposed to during their transit [54]. Namely, with both check-lists, participants were asked to indicate which of the traumatic experiences from the comprehensive lists was shared with them during their work by a person who directly experienced it. Both instruments have been shown to have good psychometric properties [42, 54].

#### Hopkins Symptom Checklist-25 (HSCL-25)

HSCL [53] consists of 25 items assessing symptoms of anxiety (10 items) and depression (15 items). The severity of symptoms is assessed using a four-point scale (1 –not at all, 2 –a little, 3 –quite a bit, and 4 –extremely). In previous studies, the scale has shown sound psychometric properties across various cultures and ethnic groups [42, 55].

#### Manchester Short Assessment of quality of life (MANSA)

The MANSA [56] assesses satisfaction with different life domains, including social relationships, safety, leisure, finances, family, accommodation, living situation, and work. It consists of 12 items scored on a seven-point Likert scale. The scale was shown to be psychometrically sound [56–58].

All procedures adhered to the Declaration of Helsinki standards. All participants signed the informed consent form prior to participating in the study, personal information was kept confidential and all the data were anonymized before conducting the analysis. The study was approved by the Institutional Review Board of Department of Psychology, Faculty of Philosophy, University of Belgrade (no. 2019–024). Questionnaires were administered online, and after filling out the questionnaires, all participants had an opportunity to be debriefed and to get individual feedback on their results.

### Data analysis

Data analysis was performed using IBM SPSS 21. Descriptive statistics were used to examine distributions of scores for the instruments used. Psychometric properties of the STSS, for both scale- and item-level analyses, were calculated using the Rtt10g macro for SPSS [59] which provided indicators of item sampling adequacy, internal consistency, homogeneity, and internal validity. The latent structure of STSS was tested with both exploratory (EFA) and confirmatory factor analysis (CFA) using maximum likelihood extraction. CFAs were performed using IBM SPSS Amos 21. To evaluate concurrent models of STS, several fit indices were consulted: χ^2^ statistic, Tucker-Lewis Fit Index (*TLI*), Comparative Fit Index (*CFI*), Root Mean Square Error of Approximation (*RMSEA*), and Standardized Root Mean Residual (*SRMR*). The following criteria for good fit were used: *TLI* and *CFI* ≥.95, *RMSEA* ≤.06, and *SRMR* ≤.08 [60] and the concurrent models were compared using the Chi-square test. Differences in the level of STS and secondary exposure to trauma between professionals providing different types of help and assistance were tested using the Analysis of Variance (ANOVA) and Bonferroni *post hoc* tests. The relations between STS and work-related measures, secondary exposure to trauma, depression- and anxiety-related symptoms, as well as the quality of life, were investigated using correlational analysis. Finally, in order to examine the integrative model of precursors and potential effects of STS, a path analysis was conducted. The evaluation of the model followed the same cut-off values as previously mentioned.

## Results

### Psychometric properties

According to Bride’s guidelines [61], 38.1% of professionals in the current sample can be considered slightly affected or completely unaffected by secondary traumatic stress symptomatology; 30.0% are mildly affected by secondary traumatic stress related difficulties; 14.4% can be considered as moderately secondary traumatized; 6.3% are suffering from highly pronounced secondary traumatic stress; and 11.1% exhibit severe secondary traumatic stress related difficulties. Using the score of 38 or above as a cutoff which indicates the need to take steps in order to address secondary traumatic stress [61], 31.9% of professionals working with refugees can be considered at risk of this syndrome.

Table 1 presents descriptive statistical measures of the three subscales of STSS as well as its global score. All three subscales exhibited positive asymmetry of distributions of scores, i.e. higher grouping around lower scores. Differences between three subscales were observed [*F*_(1.91, 513.92)_ = 14.579, *p* < .001, η_p_^2^ = .051]. Namely, participants demonstrated higher scores on arousal than on the intrusions (*p* < .001) and avoidance subscales (*p* < .001), but differences between avoidance and intrusion symptomatology were not observed.

**Table 1:** Descriptive statistics for STSS.

	*M*	*SD*	*Min*	*Max*	*Sk*	*Ku*	*K-S Z*
Intrusions	9.44	3.59	5	24	1.05	1.19	2.27**
Avoidance	13.41	4.99	7	30	0.85	0.32	2.01**
Arousal	10.28	4.10	5	24	0.75	0.16	1.77**
STSS total	33.14	11.56	17	72	0.81	0.35	1.57*

*M–*mean; *SD–*standard deviation; *Min–*minimum; *Max–*maximum; *Sk–*skewness; *Ku–*kurtosis; *K-S Z–*Kolmogorov-Smirnov test of normality;

* *p* < .05;

** *p* < .01.

The psychometric properties of the instrument were calculated using the Rtt10g macro for SPSS [59]. Item sampling adequacy was .98, indicating the high representativeness of items sampled for measuring the given construct. Reliability measures for the intrusion, avoidance, and arousal subscales were .77, .81, and .82, respectively, while the full-test internal consistency was .92. Full-test average inter-item correlation (*H1* = .39), and the proportion of variance accounted for by the first principal component relative to all reliable components (*H5* = .75) indicated high homogeneity of the instrument.

Table 2 presents the items’ internal psychometric characteristics. Item sampling adequacy for individual items varied between .97 –.99, the proportion of variance of a given item, predicted using the remaining of the test’s items (item’s reliability), was moderate for most of the items, ranging from .31 to .61. All items exhibited moderate to high positive item-total correlations ranging from .53 to .76. Similarly, all the items demonstrated moderate to high correlations with the principal object of measurement (range .54 –.76) pointing to the high internal validity of all the items.

**Table 2:** Items’ internal psychometric characteristics of STSS.

Items	rep.	rel.	internal validity	
			*H*	*B*
stss 01	.974	.333	.583	.593
stss 02	.976	.479	.644	.647
stss 03	.979	.312	.532	.541
stss 04	.981	.504	.699	.695
stss 05	.982	.566	.744	.736
stss 06	.983	.608	.758	.753
stss 07	.985	.447	.676	.673
stss 08	.986	.540	.745	.738
stss 09	.978	.437	.622	.622
stss 10	.980	.370	.596	.597
stss 11	.982	.584	.761	.755
stss 12	.979	.479	.661	.662
stss 13	.982	.375	.602	.607
stss 14	.973	.423	.610	.614
stss 15	.981	.482	.669	.665
stss 16	.980	.424	.656	.654
stss 17	.973	.317	.549	.560

rep.–item sampling adequacy; rel.–item reliability; *H*–first principal component loading; *B*–item-total correlation.

### Exploratory factor analysis

Despite the fact that the STSS was developed following DSM-4 criteria, its items partially cover the diagnostic criteria of other prominent models of PTSD. Thus, on the basis of their content, items of the STSS can be re-classified in the context of DSM-5 and other PTSD conceptualizations and their symptom clusters. Namely, 15 out of 17 STSS items in total unequivocally target diagnostic criteria for PTSD as defined in DSM-5 as well as other aforementioned models, while the remaining two items (items 5 and 9) can, depending on the model in question, be considered generic markers of negative alterations in cognitions and mood or negative affect (see Table 1).

In order to test the latent structure of STSS exploratory factor analysis (*EFA*) was conducted. Maximum likelihood was used as the extraction method and the obtained factors were rotated into the Promax position. Kaiser-Meyer-Olkin measure of sampling adequacy (.93) and Bartlett’s test of sphericity [χ^2^_(136)_ = 1986.07, *p* < .001] showed that the correlation matrix was suitable for factorization. Both Guttman-Kaiser and scree criteria suggested the retention of three factors which accounted for 48.39% of the items’ variance. Table 3 presents the results of *EFA*, alongside the classification of items according to several of the aforementioned theoretical models.

**Table 3:** Pattern matrix (maximum likelihood extraction, Promax rotation).

DSM-5 criteria	Item		*DSM-4 model*	*DSM-5 model*	*Dysphoria model*	*Dysphoric arousal*	*Anhedonia model*	*1*	*2*	*3*	*h*^*2*^
D	stss 9	I was less active than usual.	Av	NACM	Dy	NACM	NA	.**770**	-.094	-.018	.490
E1	stss 15	I was easily annoyed.	Hy	Hy	Dy	DA	DA	.**711**	-.152	.173	.527
E5	stss 11	I had trouble concentrating.	Hy	Hy	Dy	DA	DA	.**634**	.193	-.004	.598
E4	stss 8	I felt jumpy.	Hy	Hy	Hy	AA	AA	.**611**	.105	.098	.563
D6	stss 7	I had little interest in being around others.	Av	NACM	Dy	NACM	An	.**600**	-.007	.139	.473
B1	stss 10	I thought about my work with clients when I didn’t intend to.	In	In	In	In	In	.**449**	.260	-.081	.361
E3	stss 16	I expected something bad to happen.	Hy	Hy	Hy	AA	AA	.**349**	.171	.195	.394
D7	stss 1	I felt emotionally numb.	Av	NACM	Dy	NACM	An	.**316**	.050	.259	.305
E6	stss 4	I had trouble sleeping.	Hy	Hy	Dy	DA	DA	.199	.**670**	-.114	.554
B3	stss 3	It seemed as if I was reliving the trauma(s) experienced by my client(s).	In	In	In	In	In	.008	.**660**	-.104	.363
B2	stss 13	I had disturbing dreams about my work with clients.	In	In	In	In	In	-.044	.**573**	.128	.401
B5	stss 2	My heart started pounding when I thought about my work with clients.	In	In	In	In	In	-.209	.**570**	.378	.537
B4	stss 6	Reminders of my work with clients upset me.	In	In	In	In	In	-.065	.**555**	.394	.673
D	stss 5	I felt discouraged about the future.	Av	NACM	Dy	NACM	NA	.414	.**533**	-.143	.602
C1	stss 14	I wanted to avoid working with some clients.	Av	Av	Av	Av	Av	.157	-.168	.**734**	.534
C2	stss 12	I avoided people, places, or things that reminded me of my work with clients.	Av	Av	Av	Av	Av	.029	.076	.**679**	.561
D1	stss 17	I noticed gaps in my memory about client sessions.	Av	NACM	Dy	NACM	NA	.206	.031	.**367**	.292

Primary factor loadings are marked bold; *h*^*2*^ *–*communality; B criterion–intrusion symptoms associated with the traumatic event/s; C criterion–persistent avoidance of stimuli associated with the traumatic event/s; D criterion–negative alterations in cognitions and mood associated with the traumatic event/s; E criterion–marked alterations in arousal and reactivity associated with the traumatic event/s; In–intrusion symptoms; Av–avoidance symptoms; Hy–hyperarousal; NACM–negative alterations in cognitions and mood; Dy–dysphoria; DA–dysphoric arousal; AA–anxious arousal; NA–negative affect; An–anhedonia.

The first factor accounted for the variance of items from all initial subscales, but predominantly saturated items originating from the initial *arousal* and some of the *avoidance* items, i.e. the items covering D (negative alterations in cognitions and mood) and E criteria (hyperarousal and reactivity) of DSM-5 model. On the other hand, one item covering B diagnostic criteria for PTSD, specifically B1 criteria (recurrent, involuntary, and intrusive distressing memories of the traumatic event) demonstrated primary loading on this factor. Bearing in mind that this factor predominantly summarizes clusters of symptoms capturing both negative alterations in cognitions and mood, as well as hyperarousal and reactivity symptoms, this dimension is interpreted as the factor of *negative alterations in cognitions*, *mood*, *and reactivity* (NACMR). Relatively homogeneous loadings of items capturing anxious (items 8 and 16) and dysphoric arousal on the same factors (items 11 and 15) demonstrated that the structure of this factor is, contrary to the proposed models of Dysphoric and Anxious arousal, to a large extent monolithic.

The majority of items primarily generating the second factor originate from the initial *intrusion* subscale [11]. The only exceptions were items originally intended to measure hyperarousal which generally reflects sleep difficulties (item 4), and the item capturing dysphoric alteration of cognition (item 5) which exhibited relatively high loading on the first factor as well. In line with the primary loadings, this factor fairly subsumed four out of five items initially intended for measuring the B criterion of PTSD and thus can be interpreted as the *intrusion* factor.

Finally, the third extracted factor predominantly saturated two items reflecting active avoidance of stimuli and cues associated with the secondary trauma, therefore, covering the C criterion for PTSD elaborated within, both DSM-4 and DSM-5. The third item of this factor, depicting gaps in memory about client sessions, exhibited somewhat high complexity with loadings on both the third and the first factor indicating that it represents a cognitive manifestation of avoidance before negative alteration in cognition and mood, emphasizing avoidance of distressing memories, thoughts, or feelings associated with client’s trauma.

Intercorrelations between extracted factors are given in Table 4. In line with the aforementioned homogeneity indicators, extracted latent dimensions demonstrated relatively high intercorrelations, as well as fair internal consistencies.

**Table 4:** Factor correlation matrix.

**Factors**	**Dysphoria**	**Intrusions**	**Avoidance**
NACMR	.859	.669**	.598**
Intrusions		.838	.649**
Avoidance			.695

** *p* < .01; Diagonal values are measures of internal consistency (Cronbach alpha coefficients).

### Confirmatory factor analysis

Several confirmatory models of the latent structure of STS were tested. Within the first model, unity of the STS was tested, implying a single latent dimension underlying the syndrome. The second model tested the three-factor structure of secondary trauma proposed by Bride and collaborators [11] that fits the conceptualization of PTSD within DSM-4, i.e. recognizing three distinctive aspects of STS–*avoidance*, *intrusions*, and *hyperarousal* (*DSM-4 model*). The third model tested the fit of the DSM-5 conceptualization of PTSD symptomatology including four distinct clusters of symptoms–*negative alterations in cognitions and mood*, *hyperarousal*, *intrusions*, and *avoidance* (*DSM-5 model*). A four-factor model of *dysphoria*, *avoidance*, *intrusions*, and *hyperarousal* (*Dysphoria model*) underlying variations in symptomatology was also tested.

In testing all multi-facet models, we relied on both empirical findings and theoretical expectations of relatively distinct but interrelated clusters of symptoms so the covariances between factors were estimated. The maximum likelihood estimation was used in all models tested. In a single-factor model, all 17 items were allowed to load on a single dimension of STS, while in multi-factor models, items were allowed to freely load on their respective factor (see Table 1 for detailed information on the classification of all items within each model) and the loadings on other factors were set to zero. The factor correlation matrix is presented in Table 5. The summary of the tested models is given in Table 6, while the factor loadings are presented in Table 7.

**Table 5:** Factor correlation matrix.

Models	Factors	Hyperarousal	Intrusions	Avoidance
DSM-4 3-factor model	Hyperarousal			
	Intrusions	.855**		
	Avoidance	1.000**	.888**	
DSM-5 4-factor model	Hyperarousal			
	Intrusions	.849**		
	Avoidance	.726**	.821**	
	NACM	1.000**	.822**	.756**
Dysphoria 4-factor model	Hyperarousal			
	Intrusions	.842**		
	Avoidance	.728**	.822**	
	Dysphoria	.993**	.834**	.742**
Dysphoria 3-factor model	Intrusions	/		
	Avoidance	/	.791**	
	NACMR	/	.827**	.785**

** *p* < .01; NACM–negative alterations in cognitions and mood; NACMR–negative alterations in cognitions, mood, and reactivity.

Reliability measures for NACMR, intrusion, and avoidance factors were .883, .765, and .702, respectively.

**Table 6:** Model parameters.

model		*factors*	*χ*^*2*^ *(df)*	*p*	χ^2^/*df*	*TLI*	*CFI*	*RMSEA* [*90% CI*]	*SRMR*
*I*	Single-factor model	STS	349.38 (119)	< .001	2.94	.86	.88	.085 [.075–.095]	.059
*II*	DSM-4 3-factor model	Hy/In/Av	315.49 (117)	< .001	2.70	.88	.90	.079 [.069–.090]	.058
*III*	DSM-5 4-factor model	NACM/Hy/In/Av	269.17 (114)	< .001	2.36	.90	.92	.071 [.060–.082]	.055
*IV*	Dysphoria 4-factor model	Dy/Hy/In/Av	270.36 (113)	< .001	2.39	.90	.92	.072 [.061–.083]	.055
*V*	Dysphoria 3-factor model	NACM+Hy/In/Av	270.60 (116)	< .001	2.33	.91	.92	.070 [.059–.081]	.055

STS–secondary traumatic stress; Hy–hyperarousal; In–intrusions; Av–avoidance; NACM–negative alterations in cognitions and mood; χ^2^ –chi-square test, *df*–degrees of freedom; *TLI*–Tucker-Lewis/Non-normed fit index; *CFI*–Comparative fit index; *RMSEA*–Root Mean Square Error of Approximation; *SRMR–*Standardized Root Mean Residual; *TLI* ≥ .95, *CFI* ≥ .95, *RMSEA* ≤ .06; *SRMR* ≤ .08 [60].

**Table 7:** Factor loadings for tested models.

items	single-factor model	DSM-4			DSM-5				Dysphoria				Dysphoria		
		3-factor model			4-factor model				4-factor model				3-factor model		
		Av	In	Hy	NACM	In	Av	Hy	Dy	In	Av	Hy	NACMR	In	Av
stss 01	.543	.544			.546				.544				.543		
stss 02	.610		.685			.691				.691				.691	
stss 03	.505		.551			.541				.540				.540	
stss 04	.677			.661				.673	.666				.667		
stss 05	.730	.727			.729				.734				.735		
stss 06	.735		.812			.824				.825				.824	
stss 07	.651	.659			.672				.669				.668		
stss 08	.726			.741				.746				.750	.747		
stss 09	.597	.610			.635				.632				.631		
stss 10	.572		.554			.538				.538				.540	
stss 11	.741			.754				.758	.761				.760		
stss 12	.627	.621					.793				.789				.788
stss 13	.568		.612			.609				.608				.608	
stss 14	.572	.573					.683				.685				.686
stss 15	.646			.671				.665	.671				.670		
stss 16	.629			.637				.636				.640	.637		
stss 17	.513	.519			.517				.513				.513		

The single-factor model demonstrated a relatively poor fit as indicated by all fit indices. The DSM-4 three-factor model of interrelated facets of *avoidance*, *intrusions*, and *hyperarousal* exhibited a somewhat better fit [Δχ^2^_(2)_ = 33.89, *p* < .001]. On the other hand, the DSM-5-based four-factor model of *negative alterations in cognitions and mood*, *hyperarousal*, *intrusions*, and *avoidance* achieved superior fit to both of the aforementioned models [Δχ^2^_(5)_ = 80.21, *p* < .001; Δχ^2^_(3)_ = 46.32, *p* < .001]. Still, in the DSM-4 model the latent correlation between factors of *avoidance* and *hyperarousal*, as well as factors of *negative alterations in cognitions and mood* and *hyperarousal* in the DSM-5-based model achieved a value over one, i.e. the latent covariance matrix was not positive definite, which indicated over-extraction of factors. Thus, in both models, the correlation between these factors was fixed to one. Additionally, the four-factor Dysphoria model demonstrated fit superior to the first two models [Δχ^2^_(6)_ = 79.02, *p* < .001; Δχ^2^_(4)_ = 45.13, *p* < .001] with fit indices highly similar to those of the DSM-5 model [Δχ^2^_(1)_ = 1.19, *p* = .275]. However, since *dysphoria* and *hyperarousal* factors demonstrated extremely high intercorrelation, the final model that was tested was the three-factor reduced Dysphoria model in which two aforementioned factors were merged into a single factor–negative alterations in cognitions, mood, and reactivity (NACMR). Despite the fact that this model was not shown to be superior to the DSM-5 [Δχ^2^_(2)_ = 1.43, *p* = .489] and Dysphoria four-factor model [Δχ^2^_(3)_ = 0.24, *p* = .971], we have chosen this model as the most empirically suitable one since it addresses the extremely high homogeneity and latent correlations between the *dysphoria* and *hyperarousal* factors. Due to high intercorrelations and the monolithic structure of the *dysphoria/NACM* and *hyperarousal* factors, models of *Dysphoric Arousal* and *Anhedonia* were not tested.

### Precursors and the effects of STS

In addition to the type of support participants are providing, we assessed working hours (days per week spent in direct contact with beneficiaries) and the length of time spent in the field of refugee protection. The amount of direct contact with beneficiaries was not related to any of the STS factors (NACMR: *r* = .004, *p* = .949; In *r* = -.015, *p* = .808; Av *r* = -.068, *p* = .267). Similarly, STS severity proved to be uncorrelated with the length of time spent in the field of refugee protection (NACMR: *r* = .013, *p* = .827; In *r* = .017, *p* = .784; Av *r* = .018, *p* = .773).

Table 8 presents the descriptive statistics for STS factors and secondary exposure to beneficiaries’ trauma in travel and country of origin by the type of help provided.

**Table 8:** Descriptive statistics for domains of STS and secondary exposure to beneficiaries’ trauma in travel and country of origin by subgroups of service providers.

Type of aid provided	NACMR		In		Av		Trauma (in travel)		Trauma (country of origin)	
	*M*	*SD*	*M*	*SD*	*M*	*SD*	*M*	*SD*	*M*	*SD*
Legal (*N* = 49)	2.11	0.89	2.01	0.72	1.93	1.05	.77	.24	.49	.24
Psychological (*N* = 52)	2.12	0.64	1.80	0.65	1.63	0.80	.82	.18	.43	.19
Translation (*N* = 33)	2.10	0.87	1.84	0.79	1.68	0.93	.77	.29	.52	.26
Medical (*N* = 6)	1.68	0.58	1.43	0.29	1.67	0.61	.66	.26	.33	.19
Providing food and non-food items (*N* = 22)	2.09	0.78	1.82	0.62	1.59	0.84	.64	.27	.35	.23
EOR activities (*N* = 64)	1.93	0.65	1.81	0.62	1.41	0.61	.69	.24	.36	.23
Information and referrals (*N* = 44)	2.01	0.77	2.10	0.90	1.69	0.84	.46	.26	.25	.19

*M–*mean; *SD–*standard deviation; NACMR–negative alterations in cognitions, mood, and reactivity; In–intrusions; Av–avoidance.

Due to a very small number of participants who are medical professionals, this group is omitted from further analyses. No significant between-groups differences were observed for any of the STS factors [NACMR: *F*_(5, 258)_ = 0.51, *p* = .773; In: *F*_(5, 258)_ = 1.43, *p* = .215; Av: *F*_(5, 258)_ = 2.22, *p* = .053]. On the other hand, significant between-groups effects were found for the exposure to beneficiaries’ traumatic experiences in travel [*F*_(5, 258)_ = 12.52, *p* < .001, η_p_^2^ = .195] with professionals providing legal, psychological support, and those delivering EOR activities being to a greater extent exposed to these experiences (all Bonferroni *post hoc* tests *p* < .001). Similar trend-level differences were observed for professionals providing psychological support in comparison to those providing food and non-food items (*p* = .052) and EOR activities (*p* = .066).

Participants providing information and referrals were less exposed than those providing legal (*p* < .001), psychological (*p* = .002) and translation services (*p* < .001) to beneficiaries’ traumatic experiences from the country of origin [*F*_(5, 258)_ = 8.63, *p* < .001, η_p_^2^ = .143]. Moreover, people providing legal aid (*p* = .021) and translation services (*p* = .007) had more experience with traumatic stories of their beneficiaries from their country of origin than those delivering EOR activities.

In addition, no gender differences were found for any of the STS domains–NACMR [*t*_(268)_ = 1.49, *p* = .138], intrusions [*t*_(268)_ = 0.67, *p* = .504], and avoidance [*t*_(268)_ = 0.18, *p* = .857].

Descriptive statistics for measures of secondary exposure to trauma, anxiety, depression, and quality of life obtained from the whole sample are presented in Table 9. Overall, service providers were significantly more exposed to the beneficiaries’ traumatic experiences that happened during travel than those experienced in the country of origin [*F*_(1, 269)_ = 677.870, *p* < .001, η_p_^2^ = .716]. The measures of depression and anxiety expectedly demonstrated restriction of variability and grouping of scores in the lower range. Using the cut-off score of 1.75 or higher [53] 20.0% of service providers were checklist positive for anxiety, while 22.6% of them were checklist positive for major depression. On the other hand, the majority of participants reported predominant satisfaction with various aspects of their lives. All measures exhibited high internal consistencies indicated by their alpha coefficients.

**Table 9:** Descriptive statistics for secondary exposure to trauma (in travel and country of origin), anxiety, depression, and quality of life.

	*M*	*SD*	*Sk*	*Ku*	*K-S Z*	α
Trauma (in travel)	.70	.27	-0.95	0.05	2.39**	.91
Trauma (country of origin)	.40	.24	0.27	-0.70	1.09	.96
Anxiety	1.43	0.42	1.17	1.28	2.50**	.84
Depression	1.46	0.51	1.46	2.08	3.00**	.93
Quality of life	5.16	0.78	-0.28	-0.11	1.01	.87

*M–*mean; *SD–*standard deviation; *Min–*minimum; *Max–*maximum; *Sk–*skewness; *Ku–*kurtosis; *K-S Z–*Kolmogorov-Smirnov test of normality;

** *p* < .01.

Levels of depression and anxiety correlated highly (*r* = .781, *p* < .001), while both measures demonstrated moderate negative correlations with quality of life (anxiety *r* = -.551, *p* < .001; depression *r* = -.632, *p* < .001). Both measures of secondary exposure to trauma, i.e. trauma in travel (anxiety *r* = .206, *p* = .001; depression *r* = .215, *p* < .001) and trauma in the country of origin (anxiety *r* = .148, *p* = .015; depression *r* = .181, *p* = .003) were correlated with symptoms of anxiety and depression. Additionally, both measures of secondary exposure to trauma (trauma in travel *r* = -.169, *p* = .005; trauma in country of origin *r* = -.182, *p* = .003) demonstrated low negative correlations with quality of life.

Table 10 presents correlations between the three factors of STS (Dysphoria reduced 3-factor model), STSS total score, and measures of secondary exposure to trauma, anxiety, depression, and quality of life. All symptom clusters demonstrated positive correlations with secondary exposure to trauma, relatively high positive correlations with symptoms of anxiety and depression, as well as moderate negative relationships with quality of life.

**Table 10:** Correlations between secondary trauma factors, STSS total score, and external measures.

Factor	Trauma (in travel)	Trauma (country of origin)	Anxiety	Depression	Quality of life
NACMR	.238**	.180**	.718**	.753**	-.456**
Intrusions	141*	.130**	.551**	.490**	-.302**
Avoidance	.162**	.142*	.468**	.471**	-.326**
STSS	.222**	.178**	.706**	.711**	-.438**

* *p* < .05;

** *p* < .01.

Besides correlations with the quantity of client’s different traumatic experiences, factors of STS were found to be related to the specific content of those experiences. Tables in the S1 Appendix present a proportion of service providers being faced with individual traumatic experiences of their clients during travel (Table A1 in S1 Appendix) and in the country of origin (Table A2 in S1 Appendix), alongside point biserial correlations between secondary exposure to these experiences and factors of STS.

The vast majority of traumatic events from travel were shared with more than half of the professionals in the sample by their beneficiaries. The most frequently shared experience was *separation from the family*, reported by more than 90% of participants, while the request of *additional services by the smuggler* was the least frequently shared, and reported by slightly less than one-third of participants. Among traumatic experiences from the country of origin, the most frequently reported were *being forced to flee their country* and *confiscation or destruction of personal property*, which was shared with more than 80% of participants. It seems that secondary exposure to events from travel *while in prison/detention being deprived of legal rights* and *been a victim of sexual violence*, and events from the country of origin such as *forced labor*, *sexual abuse*, *witnessing burned or disfigured bodies*, *forced marriage*, and *torture* have the strongest relationship with the severity of secondary trauma related symptomatology.

In order to examine exclusive relationships between levels of STS, its precursors, and potential consequences, an exploratory path analysis was conducted. In other words, the path model was conceptualized as a cascade of psychological events consisting of several steps/stages. Namely, the quantity of different traumatic experiences being shared with service providers was specified as a precursor (the first stage in the model) of each of the STS domains (the second stage in the model). The elevation of relatively focal symptomatology in different STS domains is further specified to lead to the broader and context-unrelated depression- and anxiety-related symptomatology (the third stage in the model) which, in the end, negatively affect the overall quality of life (final stage of the model). In short, we hypothesized that the effects of secondary exposure to trauma on depression and anxiety symptoms, as well as on the quality of life, are fully mediated by STS domains. Similarly, the effects of STS domains on the quality of life are expected to be fully mediated by broader depression/anxiety symptomatology.

**Fig 1:**
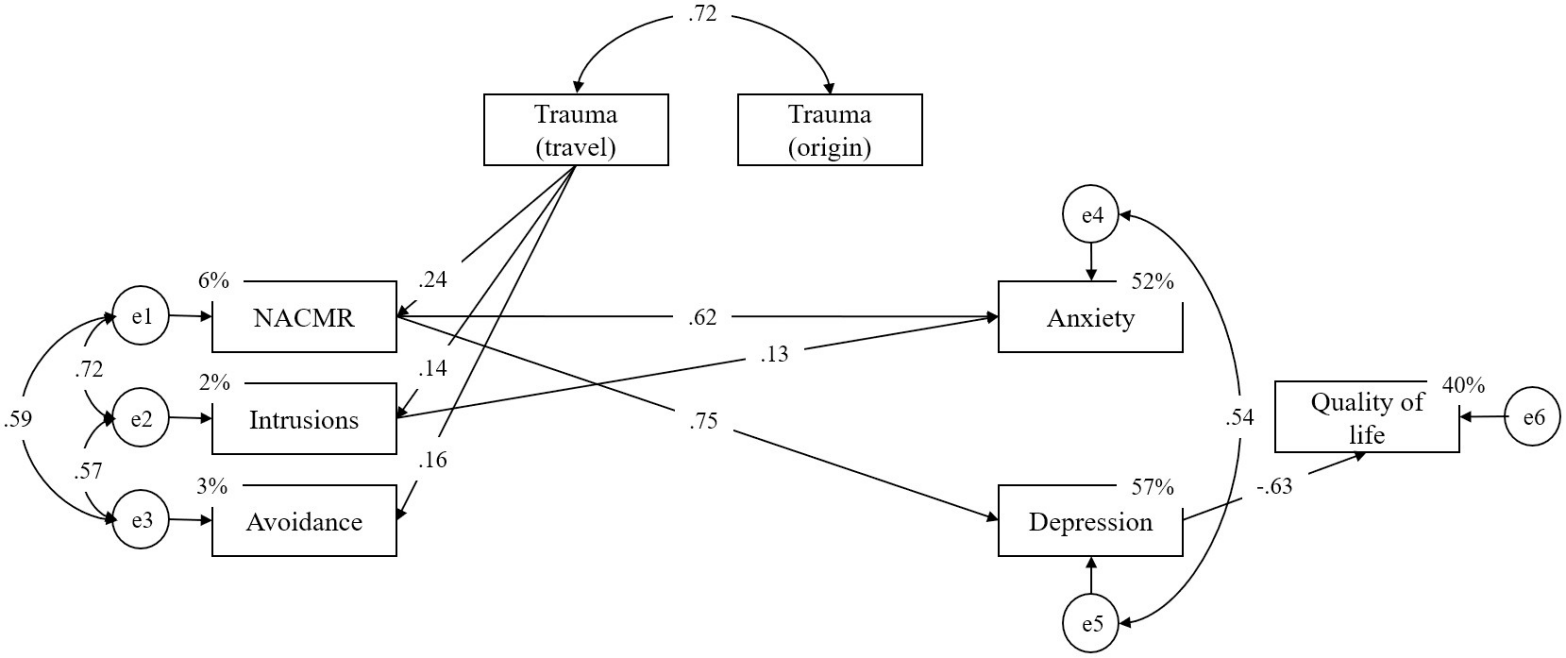
Structural relationships between STS factors, secondary exposure to trauma (client’s trauma in travel and in the country of origin), symptoms of anxiety and depression, and quality of life.

The final model, with non-significant effects fixed to zero, is depicted in Fig 1. Both relative and absolute fit indices pointed to an excellent model fit (Table 11).

**Table 11:** Path analysis parameters.

χ^2^ (df)	*p*	χ^2^/df	*TLI*	*CFI*	*RMSEA* [*90% CI*]	*SRMR*
17.53 (16)	.352	1.096	.998	.999	.019 [.000–.061]	.030

χ^2^ –chi-square test, *df*–degrees of freedom; *TLI*–Tucker-Lewis/Non-normed fit index, *CFI*–Comparative fit index; *RMSEA*–Root Mean Square Error of Approximation; *SRMR*–Standardized Root Mean Residual; *TLI* ≥ .95, *CFI* ≥ .95, *RMSEA* ≤ .06, *SRMR* ≤ .08 [60].

Two indicators of secondary exposure to traumatic events demonstrated a high correlation, but only the quantity of client’s different traumatic experiences that happened during travel to which service providers were exposed exhibited exclusive relations with all three factors of STS, while the quantity of client’s traumatic experiences from the country of origin did not show a significant relationship with any of the STS clusters of symptoms. As indicated by path coefficients, NACMR demonstrated large direct effects on anxiety as well as depression symptoms, while *intrusions* exhibited a direct effect on anxiety-related symptomatology only. On the other hand, *avoidance*-related symptomatology was not shown to have any independent direct effects on anxiety and depression. Finally, the effects of STS on quality of life were demonstrated to be fully mediated by an increase of depression-related symptomatology.

## Discussion

The current study aimed to examine the latent structure of Secondary Traumatic Stress (STS), its precursors and its effects on the well-being of professionals working in direct contact with refugees passing through the Balkan route. Professionals providing different kinds of support to migrants and refugees are faced with persons who have experienced numerous traumatic experiences and human rights violations on a daily basis [32–44] and are therefore of particular interest for studying STS. Namely, they represent the population that is the most exposed to secondary trauma since they are affected by the diverse and numerous traumatic experiences of the people with whom they interact. In other words, professionals working with refugees on a daily basis experience *“repeated or extreme exposure to aversive details of the traumatic event/s”*, therefore meeting Criterion A4 for PTSD as described by DSM-5 [21].

In this study, the STSS as one of the most utilized tools for STS assessment was used. Similarly, as in previous studies [10–13, 15, 16, 18, 19, 29], the STSS proved to have excellent psychometric properties with indicators of sampling adequacy and internal consistency exceeding .90, as well as high homogeneity. Similarly, each individual item demonstrated moderate to excellent internal psychometric properties proving that the STSS is a psychometrically sound measure.

According to Bride’s cut off score of 38 and higher [61], results showed that 31.9% of professionals in the current sample have moderate, high, or severe secondary traumatic stress-related difficulties. Similar results were obtained in studies assessing secondary trauma among nurses, indicating that 33% of study participants met the criteria for secondary traumatization [19], while in the sample of pediatric nurses, this percentage was even higher and reached 50% [18]. Furthermore, previous studies showed similar average STSS scores among different professionals, with psychologists showing somewhat less pronounced symptomatology [12, 15] in comparison to emergency nurses [19] and domestic violence therapists [13] who showed the most pronounced secondary trauma-related difficulties among different helping professions involved in the previous studies.

### The latent structure of STS

To date, only a few studies have examined the latent structure of STS and the empirical findings and conclusions they provided were inconsistent, emphasizing the need for additional evidence that will enable theoretical interpretation and understanding of empirical differences between proposed models and postulated similarities between PTSD and STS latent structures.

Since the STSS symptom coverage allows testing at least six theory-driven models of PTSD the series of performed CFA’s aimed to examine the following latent compositions of STS: 1) the unity of the STS (single-factor model); 2) the DSM-4 based three-factor model with factors of *hyperarousal*, *intrusions*, and *avoidance* [6]; 3) the DSM-5 model subsumed in the four-factor solution accompanying *negative alterations in cognition and mood*, *hyperarousal*, *intrusions*, and *avoidance* [21]; 4) the Dysphoria four-factor model [23, 24, 62] assuming factors of *dysphoria*, *avoidance*, *intrusions*, and *hyperarousal*. Since both EFC and CFA indicated that the distinction between *anxious* and *dysphoric arousal* was not supported by our data, i.e. items capturing these factors demonstrated high convergence with loading on the same factor, the model of [5] Dysphoric arousal [26] could not be tested. The same was true concerning [6] the Anhedonia model [27].

The most parsimonious and best-fitting model was the one summarizing three distinct but highly correlated factors–*intrusions*, *avoidance*, and the blend of *negative alterations in cognition*, *mood*, *and reactivity*. Contrary to our findings, previous studies that used different measures across various samples found that STS has one of the following structures two-factor [20], three-factor [11, 30, 63], four-factor [31], six-factor [4], or seven-factor[22]. These inconsistencies could be, at least partially, attributed to different samples as well as different tools used for the assessment of STS. The consistency in the number of factors and their composition is somewhat less diverse in studies that used the STSS as a tool for the assessment of secondary trauma. The majority of these studies resulted in a three-factor structure resembling DSM-4 diagnostic criteria [11, 30, 63]. However, some of the previous findings showed that extracted factors are often highly correlated [63] resulting in high convergence and blending of some of the dimensions into a single factor resulting in a two-factor solution [20]. Bearing in mind the results of the current study and the fact that none of the previous studies resulted in four or more relatively distinct factors, it seems that the maximum number of structurally distinct factors derived from the STSS is most likely limited to three. Namely, the results of the current study showed that the Dysphoria model demonstrated good fit but overall exhibited an extremely high correlation between *dysphoria* and *hyperarousal* factors pointing to insufficient differentiation between these two dimensions. One of the reasons for the low structural validity of this model could be that the two items comprising *hyperarousal* factor are not sufficient in number and in content to cover those aspects of adverse alterations in reactivity that are distinct from the variety of negative alterations in cognition and emotions underlying broader dysphoric symptomatology. However, blending of these two clusters of symptoms into a single dimension was also observed in professionals who are engaged in prolonged work with people who are affected by various psychosocial issues [4], but not in social and health care workers [11, 20, 22, 63] nor in relatives of trauma survivors [30, 31]. Therefore, the conjoint manifestation of *dysphoria* and *hyperarousal* could potentially be specific to the type of work and the relationship between professional and the recipient of their support. The question of empathic relation between professional and the recipient of support was generally overlooked in previous studies, and some authors point out the importance of carefully considering this aspect when trying to understand differences and similarities in the way secondary traumatization mimics PTSD symptomatology [22].

Bearing in mind that dysphoria can be found in a variety of mood disorders and anxious symptomatology [62] it could be expected that two focal item-markers of *hyperarousal* that reflect the core features of anxiety-related difficulties are inherently related to the broad conglomerate of dysphoric symptoms. On the other hand, if additional items following DSM-5 nomenclature capturing more diverse aspects of *hyperarousal* (but *avoidance* as well) were added, a relatively distinct factor of reactivity could be expected [22] thus potentially making the Dysphoria model of STS, similarly to the one in PTSD [25] superior to the other models.

In other words, although the STS is to a large extent mimicking PTSD symptomatology on the conceptual level it seems that the comprehensiveness of the STSS in the light of DSM-5 criteria is not sufficient, thus not allowing their identification on the empirical level.

### Traumatic experiences as precursors of STS

Secondary exposure to the traumatic events, i.e. the quantity and the quality of those experiences is a *conditio sine qua non* for STS. Previous studies, however, assessed secondary exposure to trauma as the duration of exposure to clients or patients, while specific content, i.e. the quality of that exposure was generally overlooked, even though there are studies showing the severity of secondary traumatization is related to the type of trauma, indicating that human-induced traumas cause more disruption in professionals‘ worldview then naturally caused traumas [64]. In line with this, in previous studies, exposure was conceptualized as the number of hours per week spent working with clients or patients, the number of clients or patients per day or week, and the number of years spent working with clients or patients, with no information on the content of the work itself [9]. This may be of particular importance for professionals whose job description does not necessarily include *repeated or extreme exposure to aversive details of the traumatic event/s*, as defined by the DSM-5 [21]. The question of to what extent all helping professionals involved in the previous studies meet this criterion should be raised and potentially addressed in future research in more detail since we assume this could be one of the reasons for inconsistencies in previous studies questioning the relation between exposure to traumatic content and severity of secondary trauma-related difficulties. Results on the amount of traumatic content different professionals involved in our study were exposed to is, as expected, aligned with the quality of contact different types of services require. Thus, psychologists, whose job description includes working through traumatic experiences of their clients, are to a greater extent exposed to the content of experiences from travel than those providing basic information and referrals and service providers conducting EOR activities and providing food and non-food items (trend-level differences). Moreover, legal representatives and interpreters are to a greater extent exposed to the traumatic experiences of their clients from their countries of origin than those providing basic information and referrals or those conducting EOR activities. This result is in line with expectations since legal representatives and interpreters are the ones doing preparation of beneficiaries for the asylum procedure and the ones who are present during asylum interviews and hearings which include detailed testimony and discussions on traumatic experiences from countries of origin, which represent a precondition for obtaining international protection. However, despite demonstrated differences in exposure to traumatic experiences, no significant differences in STS severity between different groups of professionals were observed. The lack of transfer of differences in secondary exposure to trauma to differences in STS severity between groups should be interpreted with caution since this study contrasted relatively small subsamples of professionals providing different types of services. As a result, the possibility of detecting potential group differences in the present study was very limited. In addition, secondary exposure to traumatizing content most certainly is not the only determinant of the STS. Namely, a variety of personality-related (e.g. individual differences in coping styles) and/or various work-related factors (e.g. received training, organizational support) that were not included in this study could potentially account for the lack of transfer of differences in secondary exposure to trauma to differences in STS severity. Future studies designed to test these hypotheses are needed.

Our results show that the quality of contact and its specific content, rather than the duration of contact, i.e. duration of daily/weekly exposure to clients or patients or total time spent in the field, is related to the severity of secondary traumatization. Specifically, our results show that experiencing a larger number of traumatic experiences is followed by an elevation in STS-related difficulties. Therefore, these findings seem to highlight the importance of proper assessment of exposure to traumatic content, as it was shown that mere duration of contact with beneficiaries does not necessarily define the amount of exposure to traumatic content, which was shown to affect the severity of secondary traumatization. This result may offer an explanation of the inconsistency between previous studies regarding the relationship between the severity of secondary traumatization and the exposure assessed as the mere duration of contact.

Additionally, some but not other traumatic experiences to which service provider was exposed seem to have an adverse effect on their well-being. Among those especially deleterious experiences are those that are severely dislocated from one’s everyday experience. Obtained correlations between individual traumatic experiences and STS are mostly low in size. This could be attributed to several formal- and content-related issues. Firstly, some of the experiences are considerably rare in the traumatized population and thus seldom transferred to the service provider; therefore, resulting in the restriction in the variance of the item which leads to the diminishing of its correlation with any external measure. Secondly, the frequency of encounters with clients that experienced the given traumatic event/s or repeated encounters with the same client who had experienced a given trauma was not accounted for within this study. Namely, in this study, the service provider was asked to report if s/he ever worked with a person who had experienced the given trauma/s without any specification of the frequency, quality, or how recent the given interaction occurred. Therefore, it is possible that the more frequent and recent encounters with person/s who have experienced certain trauma/s are cumulative and have more pronounced effects on STS. In other words, some but not all beneficiaries’ traumatic experiences may potentially have a stronger effect on some or all aspects of STS if the frequency of service provider’s encounters with specific, especially traumatizing experiences is high. Thirdly, it seems reasonable to expect that the effect of secondary exposure to traumatic content, whether isolated or cumulative, is not direct, but at least partially mediated by the ability to process and cope with the traumatized content. So individual differences in frequency of usage and the quality of certain coping mechanisms may lead to differential dealing with the content they were exposed to thus diminishing correlations between traumatic content and STS [9].

Correlations that are larger in size, as well as the higher number of significant relationships between both individual traumatic experiences and the overall quantity of different traumatic experiences and STS, were found for traumatic experiences during travel rather than in the country of origin. In other words, traumatic experiences from during travel were shown to be more predictive for STS than those experienced in the country of origin, and the extent of secondary exposure to trauma was shown to be the most predictive for NACMR. There are at least two possible reasons for this. Firstly, significantly more service providers were faced with the traumatic experiences of their beneficiaries that happened during travel thus making this cluster of secondary exposure to trauma more discriminative than the other. Secondly, it seems reasonable to assume that the type of interaction with people who experienced the given trauma/s more recently and which are more or less acutely distressed and affected by that experience will leave a deeper impact on the service providers, imposing a different type of interaction, and making him/her qualitatively more exposed and vulnerable to the transfer of traumatic content than the encounter with a person who experienced the same trauma several months or maybe years before. This result; however, could come as a surprise in light of the previous studies which showed traumatic events refugees and asylum seekers experience in their countries of origin to be more highly correlated to PTSD symptomatology, depression, and anxiety-related difficulties then traumatic events in the transit countries [42, 54]. An explanation for these inconsistencies can potentially be found in the extent to which helping professionals can more easily link themselves or feel more responsible for traumatic events refugees experience in their own or the neighboring countries compared to the countries of origin, causing exposure to this content to have a greater impact on STS. Future studies are needed to further explore this matter.

### Effects of STS

At the core of STS as a construct, similarly to PTSD, symptomatology translates to a variety of job- and socially-related activities leaving one with different functional impairments in every-day life [65]. Within the current study, we examined whether and which STS-induced psychological disturbances have an impact on closely related but distinct aspects of service providers’ mental health–namely, symptoms of depression and anxiety. Additionally, we wanted to examine the relationship between STS and service providers’ overall quality of life. Results have shown that three aspects of STS achieve differential effects on depression and anxiety-related disturbances, with NACMR being the most predictable for both clusters of symptoms, *intrusions* only impacting anxious symptomatology, and *avoidance* having no incremental effect on the severity of depressive and anxious symptomatology. The strong correlation between NACMR and depression-related symptoms points to the high transfer of negative alterations of cognition, mood, and reactivity on depression-like symptomatology which seems to be triggered by the accumulation of traumatic content. Although in the light of constraints imposed by internal consistencies of the given measures obtained correlations could be characterized as very high, it is important to note that NACMR and depression and anxiety cannot be considered the same constructs. Besides conceptual differences that are beyond the scope of this paper, there are several important notions that should be addressed. Here, depressive symptomatology should not be interpreted as a clinically significant mood deficit since the results showed that majority of the participants achieved low scores on this measure. Therefore, it seems more appropriate to speak about these deficits as ones that are depression-like. The same is true for the anxiety measure. Additionally, depression or anxiety are usually seen as disturbances not requiring a specific external reason while, in this context, they seem to be, to a large extent, triggered *by negative alterations in cognition*, *mood*, *and reactivity* as a consequence of engaging in prolonged work with traumatized people, therefore mimicking clinical manifestations of these syndromes. Finally, close relationships between broad dysphoric symptomatology that mostly contributes to NACMR and depression- and anxiety-related difficulties lies in the essence of this aspect of PTSD [62] thus high correlations between these constructs are not completely unexpected.

Besides NACMR, symptoms of anxiety seem to additionally reflect disturbances evoked by the frequency of intrusions and emerge as the output of general overflow of traumatizing content. It seems plausible that intrusion-induced difficulties are responsible for triggering more general anxiety symptoms as a reaction to the influx of uncontrollable and unwanted traumatic imagery.

Contrary to that, *avoidance* as the relatively distinct cluster of STS symptoms had no incremental value over NACMR and *intrusions* in the prediction of depression- and anxiety-related difficulties. In other words, *avoidance*-specific variance does not have an exclusive effect on the mental health of service providers but is fully mediated by dimensions of NACMR and intrusions. One of the reasons for the absence of its predictive power probably lies in the fact that the dimension of *avoidance* is represented by only two items which restricts the magnitude of its potential relationships with the outcome measures observed in zero-order correlations. However, more evidence on this matter needs to be addressed in future studies.

The results of our study showed the effects of STS domains on quality of life to be fully mediated by depression-like disturbances. On the other hand, anxious symptoms proved not to have an incremental impact on the quality of life. Therefore, it seems that STS symptoms *per se* do not have a direct but indirect impact on the quality of life by triggering more general and more pervasive mood disturbances that arise from the accumulation of secondary exposure to trauma and inability to adequately process and cope with overwhelming traumatic experiences that were communicated to service providers by people with whom they are working on a daily basis.

Finally, several additional limitations of this study should be noted. Although being out of the scope of this paper, we recognize that for a comprehensive understanding of the phenomenon of STS it is important to extensively assess the effects of both person-related as well as various work-related factors on the severity of secondary traumatization. Namely, future studies should include several factors that could contribute to STS beyond secondary exposure to trauma such as information on service providers’ mental health status and potential previous diagnoses as well as their own personal history of trauma. Additional important factors to consider include a detailed assessment of work-related factors including organizational climate and cooperation, relevant initial and continuous training received, satisfaction with salary, peer support, and support from supervisors as well as utilization and availability of psychological support services. In addition to enabling a more comprehensive understanding of protective and risk factors for secondary traumatization, the inclusion of these aspects would offer valuable guidance for the prevention of this syndrome and the protection of service providers’ mental health.

## Conclusion

The results of this study shed light on the latent composition of STS and the cascade of events that make professionals working with traumatized people especially vulnerable to STS and other psychological difficulties. The results have shown that the latent composition of the STS partially deviates from the prominent models of PTSD thus calling in question the isomorphism of these two constructs on an empirical level. The present study did not find evidence of the relationship between the amount of physical exposure to beneficiaries and the severity of STS. However, the results have shown that both the quality and the quantity of different traumatic experiences that were communicated to service providers lead to the elevation in all aspects of STS which further results in the more general anxiety-related, but predominantly depression-related disturbances that, at the end of this cascade, take a toll in the form of reducing their overall quality of life. Despite demonstrating differences in secondary exposure to traumatic experiences that proved to correspond to their job description, professionals providing different types of assistance showed no differences in the severity of STS symptoms. To fully grasp a mediating role of different factors contributing to the translation of secondary exposure to trauma into STS severity, future studies addressing both person- and work-related factors in a more comprehensive manner are needed. In the light of the current refugee crises and the largest refugee and migrant flow in the last few decades, the implications of this study should be considered and used in order to create much needed data-driven programs for training, supervision, and support aiming to protect mental health and well-being of helping professionals working with refugees all over the world.

## Supporting information

S1 Appendix Correlations between traumatic experiences and STSS. (DOCX)

## References

[1] Figley CR. Victimization, Trauma, and Traumatic Stress. Couns Psychol. 1988;16(4):635–41. 10.1177/0011000088164005.

[2] Figley CR. Compassion fatigue as secondary traumatic stress disorder: An overview. 1995; ISSN: 9780876307595.

[3] Figley, C. R. Compassion fatigue: Coping with secondary traumatic stress disorder in those who treat the traumatized. Brunner/Mazel psychological stress series, No. 23; 1995.

[4] Weitkamp K, Daniels JK, Klasen F. Psychometric properties of the Questionnaire for Secondary Traumatization. Eur J Psychotraumatol. 2014;5:1–11. 10.3402/ejpt.v5.21875 24427450 PMC3888907

[5] Elwood LS, Mott J, Lohr JM, Galovski TE. Secondary trauma symptoms in clinicians: A critical review of the construct, specificity, and implications for trauma-focused treatment. Clin Psychol Rev. 2011;31(1):25–36. 10.1016/j.cpr.2010.09.004 21130934

[6] American Psychiatric Association. Diagnostic and Statistical Manual of Mental Disorders. Fourth Edition Washington, DC, American Psychiatric Association; 2000.

[7] Hensel JM, Ruiz C, Finney C, Dewa CS. Meta-Analysis of Risk Factors for Secondary Traumatic Stress in Therapeutic Work with Trauma Victims. J Trauma Stress. 2015;28:83–91. 10.1002/jts.21998 25864503

[8] Guerra Vio C, Vidallet JLS. Psychometric examination of the Secondary Traumatic Stress Scale: a study on Chileans Professionals. Psicol Conductual. 2007;15(3):441–56.

[9] Vukčević Marković M, Živanović M. Secondary Traumatization in Service Providers working with Refugees In: Hamburger A, Hancheva C, Ozcurumez S, Scher C, Stanković B, Tutnjević S, editors. Forced Migration and Social Trauma. London and New York: Routledge 2019; 2019 p. 511–62. 10.4324/9780429432415

[10] Badger K, Royse D, Craig CD, Vegas L. Hospital Social Workers and Indirect Trauma Exposure: An Exploratory Study of Contributing Factors. Health Soc Work. 2008;33(1):63–71. 10.1093/hsw/33.1.63 18326451

[11] Bride BE, Robinson MM, Yegidis B, Figley CR. Development and Validation of the Secondary Traumatic Stress Scale. Res Soc Work Pract. 2004;14(1):27–35. 10.1177/1049731503254106.

[12] Manning-Jones S, De Terte I, Stephens M. Secondary traumatic stress, vicarious posttraumatic growth, and coping among health professionals: A comparison study. NZ J Psychol. 2016;45(1).

[13] Ben-porat A, Itzhaky H. The Contribution of Training and Supervision to Perceived Role Competence, Secondary Traumatization, and Burnout Among Domestic Violence Therapists. Clin Superv. 2011;30(1):95–108. 10.1080/07325223.2011.566089.

[14] Bride BE, Macmaster SA. Correlates of Secondary Traumatic Stress in Child Protective Services Workers. J Evid Based Soc Work. 2016 10.1300/J394v04n03_05.

[15] Makadia R, Sabin-Farrell R, Turpin G. Indirect exposure to client trauma and the impact on trainee clinical psychologists: Secondary traumatic stress or vicarious traumatization? Clin Psychol Psychother. 2017;24(5):1059–68. 10.1002/cpp.2068 28124447

[16] Devilly GJ, Wright R, Varker T. Vicarious trauma, secondary traumatic stress or simply burnout? Effect of trauma therapy on mental health professionals. Aust N Z J Psychiatry. 2009;43(4):373–85. 10.1080/00048670902721079 19296294

[17] Cornelia M. Secondary traumatic stress and posttraumatic growth: Social support as a moderator. Soc Sci J. 2016;53:14–21. 10.1016/j.soscij.2015.11.007.

[18] Kellogg MB, Knight M, Dowling JS, Crawford SL. Secondary Traumatic Stress in Pediatric Nurses. J Pediatr Nurs. 2018;43:97–103. 10.1016/j.pedn.2018.08.016 30473163

[19] Dominguez-Gomez E, Rutledge DN. Prevalence of secondary traumatic stress among emergency nurses. J Emerg Nurs. 2009;35:199–204. 10.1016/j.jen.2008.05.003 19446123

[20] Jacobs I, Charmillot M, Soelch CM, Horsch A. Validity, Reliability, and Factor Structure of the Secondary Traumatic Stress Scale-French Version. Front Psychiatry. 2019 10.3389/fpsyt.2019.00191. PMC6474258 31031651

[21] American Psychiatric Association. Diagnostic and Statistical Manual of Mental Disorders. Fifth Edition Arlington, VA, American Psychiatric Association; 2013.

[22] Mordeno IG, Go GP, Yangson-serondo A. Examining the dimensional structure models of secondary traumatic stress based on DSM-5 symptoms. Asian J Psychiatr. 2017;25:154–60. 10.1016/j.ajp.2016.10.024 28262139

[23] Watson D. Rethinking the mood and anxiety disorders: a quantitative hierarchical model for DSM-V. J Abnorm Psychol. 2005;114(4):522–36. 10.1037/0021-843X.114.4.522 16351375

[24] Watson D. Differentiating the Mood and Anxiety Disorders: A Quadripartite Model. Annu Rev Clin Psychol. 2009;5:221–47. 10.1146/annurev.clinpsy.032408.153510 19327030

[25] Yufik T, Simms LJ. A Meta-Analytic Investigation of the Structure of Posttraumatic Stress a Meta-Analytic Investigation of the Structure of Posttraumatic Stress Disorder Symptoms. J Abnorm Psychol. 2010;119(4):764–76. 10.1037/a0020981 21090877 PMC4229035

[26] Elhai JD, Biehn TL, Armour C, Klopper JJ, Frueh BC, Palmieri PA. Evidence for a unique PTSD construct represented by PTSD’s D1 –D3 symptoms. J Anxiety Disord. 2011;25(3):340–5. 10.1016/j.janxdis.2010.10.007 21094021

[27] Armour C, Tsai J, Durham TA, Charak R, Biehn TL, Elhai JD, et al Dimensional structure of DSM-5 posttraumatic stress symptoms: Support for a hybrid Anhedonia and Externalizing Behaviors model. J Psychiatr Res. 2015;61:106–13. 10.1016/j.jpsychires.2014.10.012 25479765

[28] Tsai J, Harpaz-Rotem I, Armour C, Southwick S. M., Krystal JH, Pietrzak RH. Dimensional Structure of DSM-5 Posttraumatic Stress Symptoms: results from the National Health and Resilience in Veterans Study. J Clin Psychiatry. 2015;76(5):546–553. 10.4088/JCP.14m09091 25562376

[29] Christodoulou-fella M, Middleton N, Papathanassoglou EDE, Karanikola MNK. Exploration of the Association between Nurses’ Moral Distress and Secondary Traumatic Stress Syndrome: Implications for Patient Safety in Mental Health Services. Biomed Res Int. 2017 10.1155/2017/1908712 29209622 PMC5676344

[30] Mirsaleh YR, Ahmadi K, Davoudi F, Mousavi SZ. Validity, Reliability, and Factor Structure of Secondary Trauma Stress Scale (STSS) in a Sample of Warfare Victims’ Children. Iran J Psychiatry Clin Psychol. 2014;20(2):134–43. 10.3389/fpsyt.2019.00191.

[31] Bjornestad AG, Schweinle A, Elhai JD. Measuring Secondary Traumatic Stress Symptoms in Military Spouses with the Posttraumatic Stress Disorder Checklist Military Version. 2014;202(12):864–9. 10.1097/NMD.0000000000000213. 25386765

[32] Carswell K, Blackburn P, Barker C. The Relationship Between Trauma, Post-Migration Problems and the Psychological Well-Being of Refugees and Asylum Seekers. Int J Soc Psychiatry. 2009 11 19;57(2):107–19. 10.1177/0020764009105699. 21343209

[33] Keller AS, Rosenfeld B, Trinh-Shevrin C, Meserve C, Sachs E, Leviss JA., et al Mental health of detained asylum seekers. Lancet. 2003;362:1721–3. 10.1016/S0140-6736(03)14846-5 14643122

[34] Hallas P, Hansen AR, Stæhr MA, Munk-Andersen E, Jorgensen HL. Length of stay in asylum centres and mental health in asylum seekers: A retrospective study from Denmark. BMC Public Health. 2007;7:1–6. 10.1186/1471-2458-7-288 17931414 PMC2151767

[35] Raghavan S, Rasmussen A, Rosenfeld B, Keller AS. Correlates of Symptom Reduction in Treatment-Seeking Survivors of Torture. Psychol Trauma Theory, Res Pract Policy. 2012 10.1037/a0028118.

[36] United Nations High Commissioner for Refugees [UNHCR]. Global trends: Forced displacement in 2016. Geneva, Switzerland.

[37] Priebe S, Giacco D, El-Nagib R. Public Health Aspects of Mental Health Among Migrants and Refugees: a Review of the Evidence on Mental Health Care for Refugees, Asylum Seekers and Irregular Migrants in the WHO European Region. Heal Evid Netw Synth Rep 47 2016 27809423

[38] Cantekin D, Gençöz T. Mental Health of Syrian Asylum Seekers in Turkey: The Role of Pre-Migration and Post-Migration Risk Factors. J Soc Clin Psychol. 2017;36(10):835–59. 10.1521/jscp.2017.36.10.835.

[39] Steel Z, Chey T, Silove D, Marnane C, Bryant RA, Van Ommeren M. Association of Torture and Other Potentially Traumatic Events With Mental Health Outcomes Among Populations Exposed to Mass Conflict and Displacement: a systematic Review and Meta-analysis. JAMA—J Am Med Assoc. 2009;302(5):537–49. 10.1001/jama.2009.1132 19654388

[40] Vukčević M, Dobrić J, Purić D. Mental health of asylum seekers in Serbia. Serbia, Belgrade: UNHCR; 2014.

[41] Vukčević Marković M, Gašić J, Bjekić J. Refugees’ Mental Health. Serbia, Belgrade: Psychosocial Innovation Network; 2017.

[42] Vukčević M, Momirović J, Purić D. Adaptation of Harvard Trauma Questionnaire for working with refugees and asylum seekers in Serbia. Psihologija. 2016;49(3):277–99. 10.2298/PSI1603277V.

[43] Vukčević M, Momirović J, Purić D. Study of The Mental Health of the Asylum Seekers in Serbia. UNHCR; 2014.

[44] Laban CJ, Gernaat HBPE, Komproe IH, Van Der Tweel I, De Jong JTVM. Postmigration living problems and common psychiatric disorders in Iraqi asylum seekers in the Netherlands. J Nerv Ment Dis. 2005;193(12):825–32. 10.1097/01.nmd.0000188977.44657.1d 16319706

[45] Gerritsen A a M, Bramsen I, Devillé W, van Willigen LHM, Hovens JE, van der Ploeg HM. Physical and mental health of Afghan, Iranian and Somali asylum seekers and refugees living in the Netherlands. Soc Psychiatry Psychiatr Epidemiol. 2006 1;41(1):18–26. 10.1007/s00127-005-0003-5 16341619

[46] Laban CJ, Komproe IH, Gernaat HBPE, de Jong JTVM. The impact of a long asylum procedure on quality of life, disability and physical health in Iraqi asylum seekers in the Netherlands. Soc Psychiatry Psychiatr Epidemiol. 2008;43:507–15. 10.1007/s00127-008-0333-1 18560785

[47] Steel Z, Silove D, Bird K, McGorry P. Pathways from War Trauma to Posttraumatic Stress Symptoms Among Tamil Asylum Seekers, Refugees, and Immigrants. J Trauma Stress. 1999;12(3). 10.1023/A:1024710902534. 10467553

[48] Laban CJ, Gernaat HBPE, Komproe IH, Schreuders BA, De Jong JTVM. Impact of a long asylum procedure on the prevalence of psychiatric disorders in Iraqi asylum seekers in The Netherlands. J Nerv Ment Dis. 2004;192(12):843–51. 10.1097/01.nmd.0000146739.26187.15 15583506

[49] Bogic M, Njoku A, Priebe S. Long-term mental health of war-refugees: a systematic literature review. BMC Int Health Hum Rights. 2015;15(1). 10.1186/s12914-015-0064-9 26510473 PMC4624599

[50] Kindermann D, Schmid C, Derreza-Greeven C, Huhn D, Kohl RM, Junne F, et al Prevalence of and Risk Factors for Secondary Traumatization in Interpreters for Refugees: A Cross-Sectional Study. Psychopathology. 2017;50(4):262–72. 10.1159/000477670 28704829

[51] Holmgren H, Søndergaard H, Elklit A. Stress and coping in traumatized interpreters: A pilot study of refugee interpreters working for a humanitarian organization. Interv Int J Ment Heal Psychosoc Work Couns Areas Armed Confl. 2003;1(3):22–7.

[52] Akinsulure-Smith AM, Espinosa A, Chu T, Hallock R. Secondary Traumatic Stress and Burnout Among Refugee Resettlement Workers: The Role of Coping and Emotional Intelligence. J of Traumatic Stress. 2018;31:202–12. 10.1002/jts.22279 29669182

[53] Mollica R, McDonald L, Massagli M, Silove D. Measuring Trauma, Measuring Torture. Harvard Program in Refugee Trauma; 2004.

[54] Purić D, Vukčević Marković M. Development and validation of the Stressful Experiences in Transit Questionnaire (SET-Q) and its Short Form (SET-SF). Eur J Psychotraumatol. 2019;10(1):1–11. 10.1080/20008198.2019.1611091. PMC6534217 31164967

[55] Renner W, Salem I, Ottomeyer K. Cross-cultural validation of measures of traumatic symptoms in groups of asylum seekers from Chechnya, Afghanistan, and West Africa. Soc Behav Pers. 2006;34(9):1101–14. 10.2224/sbp.2006.34.9.1101.

[56] Priebe S, Huxley PJ, Evans S. Application of the Manchester Short Assessment of Quality of Life (MANSA). Int J Soc Psychiatry. 1999;45(1):7–12. 10.1177/002076409904500102 10443245

[57] Eklund M, Sandqvist G. Psychometric properties of the Satisfaction with Daily Occupations (SDO) instrument and the Manchester Short Assessment of Quality of Life (MANSA) in women with scleroderma and without known illness. Scand J Occup Ther. 2006;13(1):23–30. 10.1080/11038120500239578 16615412

[58] Björkman T, Svensson B. Quality of life in people with severe mental illness. Reliability and validity of the Manchester Short Assessment of Quality of Life (MANSA). Nord J Psychiatry. 2005;59(4):302–6. 10.1080/08039480500213733 16195135

[59] Knežević G, Momirović K. RTT9G i RTT10G: dva programa za analizu metrijskih karakteristika kompozitnih mernih instrumenata [RTT9G and RTT10G: Two programs for the analysis of metric properties of composite measuring instruments]. In: Kostić P, editor. Merenje u psihologiji 2. Belgrade: Institute for Criminological and Sociological Research; 1996;35–56.

[60] Hu LT, Bentler PM. Cutoff criteria for fit indexes in covariance structure analysis: Conventional criteria versus new alternatives. Struct Equ Model. 1999;6(1):1–55. 10.1080/10705519909540118.

[61] Bride BE. Prevalence of secondary traumatic stress among social workers. Soc Work. 2007;52(1):63–70. 10.1093/sw/52.1.63 17388084

[62] Simms LJ, Watson D, Doebbeling BN. Confirmatory Factor Analyses of Posttraumatic Stress Symptoms in Deployed and Nondeployed Veterans of the Gulf War. J Abnorm Psychol. 2002;111(4):637–47. 10.1037/0021-843X.111.4.637 12428777

[63] Bride BE, Ting L, Jacobson JM, Bride BE, Harrington D. The Secondary Traumatic Stress Scale (STSS): Confirmatory Factor Analyses with a National Sample of Mental Health Social Workers. 2005. 10.1300/J137v11n03_09.

[64] Cunningham M. Impact of trauma work on social work clinicians: Empirical findings. Soc Work. 2003;48(4):451–459. 10.1093/sw/48.4.451 14620102

[65] Gołąb A, Partyka M, Rzeszutek M. Secondary traumatic stress among psychotherapists: determinants and consequences. Euh-EEduPl. 2016.

