# Supplementary material for: Latent structure of secondary traumatic stress, its precursors, and effects on people working with refugees
Source: PLoS One. 2020 Oct 30;15(10):e0241545. doi: 10.1371/journal.pone.0241545 (PMC7598499; doi:10.1371/journal.pone.0241545)
Supplement: S1 Appendix — (DOCX) [file pone.0241545.s001.docx]

**S1 Appendix**

**Table A1. Correlations between individual clients’ traumatic experiences in travel and factors of STS and STSS total score.**

| **Traumatic experience during travel** | **p** | **NACMR** | **In** | **Av** | **STSS** |
| --- | --- | --- | --- | --- | --- |
| Getting lost | .69 | **.152*** | **.141*** | **.133*** | **.162**** |
| Lack of shelter | .74 | **.178**** | .095 | **.172**** | **.170**** |
| Lack of food or water | .78 | **.158**** | .054 | .113 | **.136*** |
| Suffer a serious bodily injury | .79 | **.135*** | .094 | **.120*** | **.134*** |
| Life threatened | .67 | **.152*** | **.134*** | .051 | **.148*** |
| Someone close to them dies | .81 | **.150*** | .092 | .034 | **.131*** |
| Separation from family/friends | .93 | .117 | .104 | .099 | **.123*** |
| Personal property or money was taken from him/her illegally or violently | .82 | **.125*** | .018 | .035 | .092 |
| Been a victim of discrimination | .83 | **.160**** | .071 | .079 | **.137*** |
| Been a victim of psychological violence | .84 | .106 | .046 | .087 | .095 |
| Been a victim of physical violence | .86 | **.136*** | .092 | .113 | **.134*** |
| Been a victim of sexual violence | .41 | **.197**** | **.126*** | **.159**** | **.191**** |
| Smuggler did not fulfill the deal | .79 | **.131*** | .049 | .099 | .115 |
| The smuggler requested additional services | .29 | **.138*** | .110 | **.138*** | **.144*** |
| To be detained or imprisoned | .81 | .118 | .076 | .112 | .116 |
| While in prison/detention being deprived of legal rights | .49 | **.228**** | **.140*** | **.150*** | **.214**** |
| While in prison/detention was he/she deprived of basic living conditions | .46 | **.135*** | .038 | .114 | .116 |
| Deportation | .66 | **.176**** | **.172**** | .054 | **.176**** |
| Been deprived of the relevant information by the police | .61 | **.130*** | .020 | .068 | .100 |

p – the proportion of service providers being faced with a given traumatic experience by their client in travel; NACMR – negative alterations in cognition, mood, and reactivity; In – intrusions; Av – avoidance; ** p* < .05; *** p* < .01

**Table A2. Correlations between individual clients’ traumatic experiences in the country of origin and factors of STS, and STSS total score.**

| **Traumatic experience in the country of origin** | **p** | **NACMR** | **In** | **Av** | **STSS** |
| --- | --- | --- | --- | --- | --- |
| Lack of shelter | .73 | .051 | .104 | .115 | .082 |
| Lack of food or water | .58 | .001 | .033 | .026 | .015 |
| Been without access to medical care | .71 | .021 | .003 | .016 | .017 |
| Confiscation or destruction of personal property | .84 | .054 | .009 | .051 | .046 |
| Witnessed shelling, burning, or razing of residential areas or marshlands | .74 | .113 | .085 | **.151*** | **.122*** |
| Witnessed chemical attacks on residential areas or marshlands | .20 | .103 | **.142*** | **.153*** | **.133*** |
| Exposed to minefield, blasting buildings or vehicles | .49 | **.121*** | .112 | .099 | **.128*** |
| Gathering the wounded or the dead | .38 | **.167**** | .027 | **.125*** | **.135*** |
| Exposure to frequent and unrelenting sniper fire | .39 | .045 | -.052 | -.042 | .007 |
| Used as a human shield | .09 | .106 | .099 | **.137*** | .119 |
| Combat situation (e.g. shelling and grenade attacks) | .64 | **.124*** | .036 | .052 | .099 |
| Serious physical injury from combat situation or landmine | .56 | .103 | **.131*** | **.148*** | **.129*** |
| Participated in combat missions | .51 | **.142*** | .073 | .094 | **.129*** |
| Family member or close friend participated in combat missions | .67 | .102 | .100 | .039 | .103 |
| Beating to the body | .71 | **.160**** | .090 | .108 | **.148*** |
| Attack with cold weapon | .57 | .086 | .069 | .092 | .091 |
| Torture, i.e., while in captivity he/she received deliberate and systematic infliction of physical or mental suffering | .57 | **.193**** | **.193**** | .104 | **.201**** |
| Sexual abuse | .34 | **.214**** | **.133*** | **.175**** | **.206**** |
| Rape | .34 | **.143*** | **.122*** | **.175**** | **.156*** |
| Rape by an enemy army (groupation, troop, etc.) | .11 | .016 | .002 | -.005 | .010 |
| Forced to provide sexual favours to soldiers in the ”holy war“ | .03 | .028 | -.035 | -.007 | .007 |
| Pregnancy as a result of a rape | .13 | .110 | .055 | .081 | .100 |
| Forced prostitution | .11 | .083 | .065 | .079 | .086 |
| Kidnapped/ taken hostage | .27 | .074 | .063 | .085 | .080 |
| Disappearance or kidnapping of a spouse or a spouse taken as a hostage | .51 | .102 | .117 | .084 | .115 |
| Disappearance or kidnapping of a child or a child taken as a hostage | .31 | .020 | .056 | .054 | .038 |
| Family member (child, spouse, etc.) disappeared, kidnapped or taken as a hostage | .56 | .103 | .068 | .071 | .098 |
| Rape of a family member or a friend | .19 | -.010 | .064 | .058 | .022 |
| Murder, or death due to violence of spouse | .56 | **.136*** | .105 | **.140*** | **.142*** |
| Murder, or death due to violence of child | .29 | .116 | .066 | .107 | .112 |
| Murder, or death due to violence of other family member or friend | .69 | **.143*** | .051 | .043 | .115 |
| Received the body of a family member (child, spouse, etc.) and prohibited from mourning them and performing burial rites | .29 | .070 | .009 | .033 | .053 |
| Witness beatings to head or body | .64 | .103 | .006 | .035 | .074 |
| Witness rape or sexual abuse | .18 | .012 | .076 | .056 | .039 |
| Witness torture | .41 | .022 | .035 | .004 | .026 |
| Witness mass execution of civilians | .16 | .022 | -.044 | -.022 | -.003 |
| Witness burned or disfigured bodies | .37 | **.223**** | **.150*** | .075 | **.203**** |
| Forced to destroy someone else's property or possessions | .11 | .091 | .029 | -.012 | .066 |
| Forced to betray someone who is not family or friend placing them at risk of death or injury | .09 | .082 | .056 | .047 | .077 |
| Forced to betray family member, or friend placing them at risk of death or injury | .09 | .109 | .092 | .050 | .107 |
| Forced to physically harm someone who is not family or friend | .13 | **.178**** | **.177**** | .069 | **.180**** |
| Forced to physically harm family member or friend | .07 | .061 | .071 | .069 | .071 |
| Killed someone | .13 | .072 | .067 | .062 | .077 |
| Someone was forced to betray him/her and place him/her and their family at risk of death or injury | .15 | .073 | .076 | .040 | .077 |
| Extortion or robbery | .46 | .104 | .013 | .088 | .085 |
| Searched | .57 | .082 | .026 | .016 | .063 |
| Present while someone searching for people or things in their home (or in place where he/she were living) | .44 | **.152*** | .093 | .075 | **.138*** |
| Witnessed the desecration or destruction of religious shrines or places of religious instruction | .30 | .026 | .021 | -.049 | .016 |
| Witnessed the arrest, torture, or execution of religious leaders or important members of tribe | .23 | .066 | .066 | .025 | .067 |
| Imprisonment | .50 | .116 | .090 | **.155*** | **.126*** |
| Solitary confinement | .27 | -.008 | .019 | .092 | .014 |
| Imprisonment without hygienic conditions, possibility to move, sleep, use the toilet, etc. | .39 | .108 | **.133*** | .054 | **.120*** |
| Brainwashing | .20 | .017 | .056 | .059 | .037 |
| Forced recruitment | .44 | **.166**** | **.201**** | .115 | **.187**** |
| Forced marriage | .40 | **.179**** | **.171**** | **.228**** | **.203**** |
| Forced labor | .36 | **.240**** | **.168**** | **.233**** | **.243**** |
| Victim of organ trafficking | .11 | .023 | .024 | -.052 | .015 |
| Family member or friend victim of organ trafficking | .09 | .041 | .057 | -.002 | .044 |
| Confined to home because of danger outside/Forced to hide | .68 | .109 | .019 | .071 | .087 |
| Unable to gain support from local authorities for physical or emotional problems | .69 | **.171**** | .037 | .050 | **.130*** |
| Expelled from country due to ethnic origin, religious affiliation or sexual orientation | .64 | .055 | .014 | **.136*** | .060 |
| Forced to leave their hometown and settle in a different part of the country with minimal services | .67 | **.181**** | **.131*** | **.180**** | **.184**** |
| Forced to flee their country | .89 | **.129*** | .032 | .117 | .111 |

p – the proportion of service providers being faced with a given traumatic experience by their client in travel; NACMR – negative alterations in cognition, mood, and reactivity; In – intrusions; Av – avoidance; ** p* < .05; *** p* < .01
